# Fostering positive mental health outcomes: exploring the impact of internal cohesion psychotherapy on self-esteem, self-regulation, and motivation among youth with mood disorders

**DOI:** 10.1186/s40359-025-03512-3

**Published:** 2025-11-12

**Authors:** Fitim Uka, Lum Zharku, Rineta Maliqi, Mojtaba Habibi Asgarabad, Dorentina Podrimqaku, Arian Musliu, Ayse Naz Acik, Oleksandra Loshenko, Regina Fernández Morales, Vanesa Sopjani, Angjelina Krasniqi, Klea Vezaj

**Affiliations:** 1 University of Prishtina, Prishtina, Republic of Kosovo; 2 Norwegian University of Science and Technology (NTNU), Trondheim, Norway; 3 Departmentof Psychology, Istinye University, Istanbul, Turkey; 4 Instituti ATOM, Prishtina, Republic of Kosovo; 5 University of Prishtina, Prishtina, Republic of Kosovo; 6 University of Padua, Padua, Italy; 7 Taras Shevchenko National University of Kyiv, Kyiv, Ukraine; 8 Universidad Francisco Marroquín, Guatemala City, Guatemala; 9 University of Augsburg, Augsburg, Germany; 10 University for Business and Technology, Prishtina, Republic of Kosovo

**Keywords:** Internal Cohesion Psychotherapy (ICP), Self-regulation, Self-esteem, Motivation, Youth mental health, Mood disorders

## Abstract

This study investigates the effectiveness of Internal Cohesion Psychotherapy (ICP) in enhancing three dimensions of the intrapersonal system: self-regulation, self-esteem, and motivation among youth with diagnosed mood disorders. Using a mixed-methods approach, quantitative data were collected through pre- and post-intervention assessments from 82 clients (63 females, 19 males). In contrast, qualitative data from semi-structured interviews with 20 clients (15 females, 5 males) provided deeper insights into participants’ experiences. Results from linear mixed models showed significant improvements in self-regulation, self-esteem, and motivation of the youth clients after the Internal Cohesion Psychotherapy treatment that they received. Qualitative findings underscored the practical and emotional transformations facilitated by ICP, revealing some important features of this therapeutic approach, which positively influenced emotional regulation, self-confidence, and motivation, thus improving the overall intrapersonal system. This research highlights the potential of ICP to promote positive mental health outcomes by addressing intrapersonal dimensions.

## Background

Positive mental health is more than the absence of mental illness; it encompasses the presence of psychological well-being [1]. While addressing the negative aspects of health is crucial, a sole focus on deficits can overlook the potential for fostering strengths and capacities within individuals [2]. Fostering positive mental health outcomes shifts the focus from merely alleviating distress to cultivating resilience, optimism, and personal growth. To alleviate the burden of common mental disorders, a novel line of research suggests that it might be fruitful to monitor and promote positive mental health in people with a mental disorder as a complement to monitoring and treating clinical symptoms [3–8]. This broader perspective not only reduces the likelihood of relapse but also equips individuals with tools to thrive in various aspects of life. Internal Cohesion Psychotherapy (ICP), a novel integrative psychotherapy, demonstrates this approach, emphasizing that improving positive psychological qualities and experiences can significantly improve a client’s overall well-being [9].

## The positive aspects of mental health and mood disorders

The two continua model of mental health postulates that mental illness and positive mental health are two related but independent continua [7, 10–12]. This means that a person can have a full-blown mental disorder in combination with a low level of positive mental health, but it can also mean that a person with a full-blown mental disorder has an above-average level of positive mental health [12–16]. Positive mental health entails emotional well-being (feeling joyful), social well-being (contributing to society and maintaining healthy social relationships), and psychological well-being (personal growth, self-acceptance, and purpose in life) [15]. While psychological well-being is integral to positive mental health, it is also a distinct construct, originally developed by [17], who identified six key dimensions: self-acceptance, positive relations with others, autonomy, environmental mastery, purpose in life, and personal growth. Both constructs are essential for understanding well-being, but differ in their conceptual focus and scope. Flourishing people have high emotional, social, and psychological well-being [6, 18, 19]. Conducted a meta-analysis showing that positive psychology interventions, such as cultivating gratitude and optimism, significantly improve well-being and reduce depressive symptoms. This suggests that the promotion of positive mental health is more beneficial than concentrating exclusively on the treatment of mental disorders.

Mood disorders are among the earliest identified mental health conditions, and recent advancements in epidemiological research, informed by diagnostic frameworks such as the ICD-10 and DSM, have significantly improved our comprehension of their prevalence and impact [20]. For instance, major depressive disorder (MDD) is characterized by symptoms such as persistent sadness, diminished interest in activities, fatigue, and disruptions in sleep and appetite, often accompanied by impaired concentration and feelings of worthlessness [21]. Bipolar disorder, on the other hand, involves alternating manic and depressive episodes, with manic phases featuring elevated mood, rapid speech, and impulsivity, while depressive episodes resemble those of MDD [22].

Persistent depressive disorder, formerly referred to as dysthymia, is characterized by chronic low mood and energy persisting for at least two years. In contrast, cyclothymic disorder is distinguished by cycles of mild depressive and hypomanic symptoms that disrupt daily functioning [23]. Anxiety disorders are defined by a variety of symptoms that can considerably hinder everyday living, such as persistent worry, restlessness, fatigue, difficulty concentrating, irritability, muscle tension, and sleep disturbances [21].

These mood disorders are notably prevalent, particularly among youth. Globally, MDD affects approximately 280 million people, with 10–20% of adolescents experiencing mental health conditions annually [24, 25]. Bipolar disorder affects about 1–2% of the population, with symptoms typically emerging in adolescence and a higher risk in females [26]. Moreover, significant recurrence rates highlight the necessity of effective therapies, as untreated MDD recurs in 60–80% of patients, and episodes of bipolar disorder are frequently triggered by stress or nonadherence to interventions [26, 27]. Conversely, anxiety disorders are the most prevalent mental health condition among adolescents, affecting 4.4% of individuals aged 10–14 years and 5.5% of those aged 15–19 years [28]. If left untreated, mood disorders can significantly impact social, academic, and professional performance, frequently resulting in chronic or episodic courses [29]. The necessity of specialized treatments like Internal Cohesion Psychotherapy (ICP) is thus highlighted.

## What is internal cohesion psychotherapy?

ICP is a novel approach from the Theory of Internal Cohesion [30]. This approach integrates established psychological theories and concepts, combining and expanding them to gain a deeper understanding and more comprehensive explanation of mental health. Making it an integrative approach that addresses the gaps of inefficiency left by monotherapies [31]. First, it supports the biopsychosocial model of psychopathology [32], which emphasizes the role of biological, psychological, and social factors in the progression and maintenance of psychopathology. However, while ICP acknowledges the role of biological factors, it primarily focuses on the areas that the client can influence and improve, which in this case are the psychological and social factors [33]. Like cognitive behavioral approaches, ICP considers both the event and how it is perceived (e.g., thought process) as factors that may impact internal cohesion. According to [34], most people who seek psychological help struggle to perceive circumstances objectively and have a tendency to think distortedly, which affects both intrapersonal and interpersonal relationships. ICP follows the person-centred tradition, placing the client’s needs and experiences at the core of therapy [35]. From psychoanalysis, it adopts the view that unspoken or suppressed content can create difficulties, making open communication essential [36, 37]. Lastly, ICP draws on dynamic systems theories, which stress the interrelated nature of human functioning across systems [38]. But unlike approaches that focus on a single factor, ICP emphasizes that the relations between systems are crucial, such that development or change in one area inevitably influences the others [33, 39].

This approach views individuals through four main dimensions: intrapersonal, interpersonal, professional, and spiritual. Combined, these systems offer a holistic view of the whole human experience, due to their interconnected nature [33, 39]. ICP uses its systems, specifically, the more established and positive ones, to improve clients’ negative symptoms or causes of distress. In addition, the temporal perspective is given special attention by addressing past, present, and future thoughts and emotions. The intrapersonal relationship focuses on the individual’s relationship with themselves. This relationship includes their self-esteem, self-regulation, and motivation. An established intrapersonal system is characterized by honest self-communication and self-acceptance​. The interpersonal system, on the other hand, includes relationships with others, such as with family, friends, and broader social networks. Supportive and healthy relationships with empathy and understanding are crucial for this system. The main aspects of the professional system include individuals’ careers, academic levels, and life goals and aspirations. The goal of this system is to maintain a sense of purpose by aligning inner goals with people’s profession or their academic journeys. Lastly, the spiritual system includes a person’s search for meaning and purpose in life and their religious (or lack thereof) practices. The primary goal of ICP is to achieve internal cohesion by harmonizing the individual’s relationships within and across the four systems while integrating their past, present, and future experiences [33, 39]. By addressing these dimensions, ICP promotes resilience, psychological health, and a balanced life perspective.

## Internal cohesion psychotherapy and positive youth development

Positive Youth Development (PYD) was developed in the 1990 s due to a convergence of interests in resilience, the strengths of youth, and the flexible nature of human development [40]. PYD is a strength-based view of adolescence that emphasizes how young people may flourish in supportive environments and social interactions [41]. This view is part of a broader movement in psychology that focuses on positive and supportive aspects, with multiple theories and approaches like positive psychology interventions, mindfulness-based programs, and acceptance and commitment therapy sharing PYD’s core goals [42–44]. These approaches have shown encouraging results in recent trials, particularly among teenagers. Two concepts that have shown promise in adult and adolescent therapy are the concept of best possible selves and gratitude [43]. Along these lines, positive psychology interventions aim to increase well-being by teaching various skills such as gratitude, savouring, and adaptive resources instead of focusing on strategies to reduce negative or maladaptive strategies [42]. Additionally, mindfulness-based interventions have been shown to improve self-control, emotion regulation, and executive, behavioral, and socioemotional skills in addition to boosting subjective well-being and lowering stress, anxiety, and depression [44]. With increased awareness, interventions have not only developed further but have also begun to integrate different techniques from acceptance and commitment therapy (ACT) and dialectical behavior therapy (DBT) [44]. The importance of PYD, which has become a preeminent framework for youth development, is further supported by the success of positive interventions more generally. PYD has shown extensive benefits for behavioral health and development and has been used as a framework for youth programs in high-income nations [45]. Nonetheless, its effectiveness can also be seen in different social contexts, such as countries with low or medium income. Specifically, out of the 35 programs in low-income countries that underwent thorough reviews, 21 showed improvements in behaviors, including substance abuse and hazardous sexual behavior, as well as more distant developmental outcomes like health and employment indicators through PYD interventions [46]. Similarly, studies looked at young people’s strengths and their relationship with the resources available in Kosovo, and they found that PYD’s principles are in line with the youth there [47, 48]. Recent research has demonstrated a promising connection between Internal Cohesion Psychotherapy (ICP) and Positive Youth Development (PYD) in supporting young people struggling with anxiety and depression [49]. This connection was primarily seen between PYD’s internal assets, like commitment to learning and positive identity, and ICP’s intrapersonal aspects, such as motivation and self-esteem. As well as external assets like support, boundaries, and expectations with ICP’s interpersonal aspects, such as family, friends, and romantic relationships. Furthermore, another study [39] employed a qualitative approach to examine clients’ experiences with ICP. It confirmed its effectiveness in enhancing mental well-being by empowering clients’ positive aspects and challenging their negative thought patterns. Both studies emphasize how important external support networks, such as family and the community, are for boosting the therapeutic benefits. Crucially, ICP’s fundamental tenets stress that although people have some influence over their internal communication, their interpersonal, professional, and spiritual aspects have a significant impact on their overall well-being. This emphasizes how important it is to create a supportive atmosphere to reap the rewards of therapeutic interventions completely.

## The current study

Building upon prior research demonstrating a link between Internal Cohesion Psychotherapy (ICP) and Positive Youth Development (PYD) (e.g., [39, 49]), this study investigates the effectiveness of ICP in enhancing three key aspects of positive mental health in youth with diagnosed mood disorders: motivation, self-regulation, and self-esteem. This study employs a mixed-methods approach, incorporating both quantitative and qualitative research methods. Evaluating the client’s progress from psychotherapy using a pre- and post-intervention design as well as thematic analysis of clients’ interviews. Being the first to do so in the context of psychotherapy research in tandem with PYD. Consistent with previous qualitative findings (e.g., [39, 49]), we hypothesize that participants will exhibit significant improvements in motivation, self-regulation, and self-esteem following the intervention.

## Method

### Participants

The study sample comprised 82 clinically diagnosed clients (63 females, 19 males) coming from various regions of Kosovo and receiving psychotherapy services at the *Empatia* clinic in Prishtina. Clients were diagnosed by their psychotherapists using the DSM-5 criteria, and the diagnoses included depressive disorders, anxiety disorders, post-traumatic stress disorder (PTSD), obsessive-compulsive disorder (OCD), and adjustment disorder. Clients completed a minimum of four sessions of ICP, irrespective of whether the treatment was fully completed. The mean number of ICP sessions attended was seven, and the sample was divided into three main age groups: 25 to 34 years (*n* = 60), 19 to 24 years (*n* = 18), and 14 to 18 years (*n* = 4). Most participants (*n* = 45) indicated holding a bachelor’s degree, while 25 stated they had completed a master’s degree or higher. Additionally, two participants reported completing elementary/middle school, and ten completed high school. When controlling for age, the educational level correlated with the participant’s age, as younger participants are more likely to have lower education levels, which is expected for the age group. Regarding employment status, most participants were employed full-time (*n* = 49). Seventeen participants were unemployed, thirteen held part-time jobs, and three reported volunteering.

The qualitative study sample consisted of 20 participants (15 females and 5 males), with a mean age of 25.68 years (SD = 4.75). All individuals who completed the quantitative phase were invited to take part in the qualitative component, with the first 20 providing informed consent included in the sample. Regarding educational background, 14 participants held a bachelor’s degree, 3 had completed a master’s degree, and 3 had finished high school. To ensure homogeneity in psychological readiness and engagement with the therapeutic process, exclusion criteria like severe cognitive impairment, acute psychotic symptoms, or any condition that could significantly limit participants’ ability to participate in the intervention were applied. The sample size of 20 was deemed adequate for conducting in-depth qualitative analysis and achieving data saturation, consistent with established recommendations [50] and comparable studies exploring psychotherapy processes [51].

### Procedure

For the quantitative part, participants underwent assessments at the beginning of the intervention and after a minimum of four ICP sessions, using three questionnaires: The Internal Cohesion Questionnaire, the Rosenberg Self-Esteem Scale, and the Short Self-Regulation Questionnaire. Before each assessment, participants were guided on completing the questionnaires, and the importance of providing honest and reflective responses was emphasized. Online data collection was done using the sosciurvey platform to facilitate ease of participation and ensure confidentiality.

For the qualitative study, participants were interviewed once using a semi-structured interview protocol based on ICP principles. Interviews lasted approximately 1 h and took place between April 2022 and March 2024. Two trained psychology practitioners conducted the interviews in Albanian, in a quiet room at the clinic to minimize distractions and provide a comfortable environment. Before the interview, participants were informed about the study’s purpose and their rights and provided with an informed consent form. Upon their consent, all interviews were recorded to facilitate accurate transcription and analysis.

 Ethical approval for the study was obtained from the ethical committee of the multidisciplinary clinic “Empatia.” All procedures involving human participants were conducted in accordance with the ethical standards of the committee and with the principles outlined in the Declaration of Helsinki. Secure access to assessment platforms was provided to participants, ensuring the confidentiality and privacy of their responses. Throughout the study, rigorous adherence to ethical guidelines was maintained to protect participant rights and well-being. A comprehensive monitoring process was implemented to ensure data integrity and address any issues or concerns. 

### Measures

This study administered three main instruments to assess participants’ internal cohesion, self-esteem, self-regulation, and motivation. First, the *Internal Cohesion Questionnaire* (ICQ) [38] was used to assess internal cohesion across four systems (intrapersonal, interpersonal, professional, and spiritual) within the ICP framework. It consists of 50 items rated on a 5-point Likert scale (0 = *never* to 4 = *very often*). Internal consistency for each subscale ranged from 0.73 to 0.89. The Subscale of ICP was used to assess the motivation of the clients. Next, the *Rosenberg Self-Esteem Scale* [52] is a 10-item scale assessing self-esteem. Participants rate items using a 4-point Likert scale (1 = *strongly disagree* to 4 *= strongly agree*). The RSS has a minimum score of 10 and a maximum score of 40. In the present study, internal consistency was 0.84. *Short Self-Regulation Questionnaire* (SSRQ) [53] is a self-reported questionnaire, based on the Self-Regulation Questionnaire (SRQ) [54]. This measure was used to assess self-regulation across seven processes. Participants rate items on a 5-point Likert scale (1 = *strongly disagree* to 5 *= strongly agree*). The SSRQ has a single factor representing overall self-regulation capacity. Internal consistency for the overall scale was 0.79. Lastly, to capture participants’ perspectives on changes within the intrapersonal system, an interview guide grounded in the principles of Internal Cohesion Psychotherapy (ICP) was developed. The guide followed a semi-structured format, with questions serving as flexible prompts rather than a fixed script, allowing for deeper exploration of participants’ experiences. To trace the evolution of these changes, the intrapersonal system was explored across three temporal dimensions: past, present, and future.

### Statistical analysis

The three continuous variables of interest (self-regulation, self-esteem, and motivation) of this study were measured at two time points: the past (before intervention) and the present (during the intervention) to assess changes over time that could be influenced by the intervention. For descriptive statistics, we computed the means and standard deviation of all three variables for both time points. To see the impact of the ICP approach on self-esteem, self-regulation, and motivation while controlling for potential confounding factors (age, gender, education, and job status), we employed Linear Mixed Models (LMM). These models included time (past vs. present) as a fixed effect, with participant ID as a random intercept to account for the repeated measurements across time. Additionally, age, gender, education, and job status were included as fixed effects. To complement the LMM results, effect sizes were calculated using Cohen’s d (d) to quantify the magnitude of changes between the past and present timeframes. Cohen’s d values were interpreted using standard conventions (small: d = 0.2, medium: d = 0.5, large: d = 0.8). Regarding the qualitative part, we used thematic data analysis to examine the findings from 20 interviews following [55] guidelines. The intrapersonal system components: self-regulation, self-esteem, and motivation, were treated as predefined themes in the analysis. Within each theme, subthemes were inductively derived based on participants’ narratives, resulting in two subthemes per main theme that captured the specific ways therapy influenced these aspects of the intrapersonal system. The coding process was conducted manually by two independent researchers. Each began by coding the data separately, after which they compared, refined and agreed on themes to ensure consistency. An audit trail was maintained to capture coding decisions, thematic development, and reflections throughout the process. Data saturation was judged to be achieved when no new codes or subthemes appeared, which occurred by the 18th interview.

## Results

This section looks at the effects of Internal Cohesion Psychotherapy (ICP) on 3 intrapersonal dimensions. We categorize our findings into 3 subsections: self-regulation, self-esteem, and motivation. Each subsection includes quantitative results derived from the LMM and qualitative insights from the thematic analysis of client interviews regarding the therapy’s effects.

### Growth in self-regulation after treatment with ICP

**Fig. 1:**
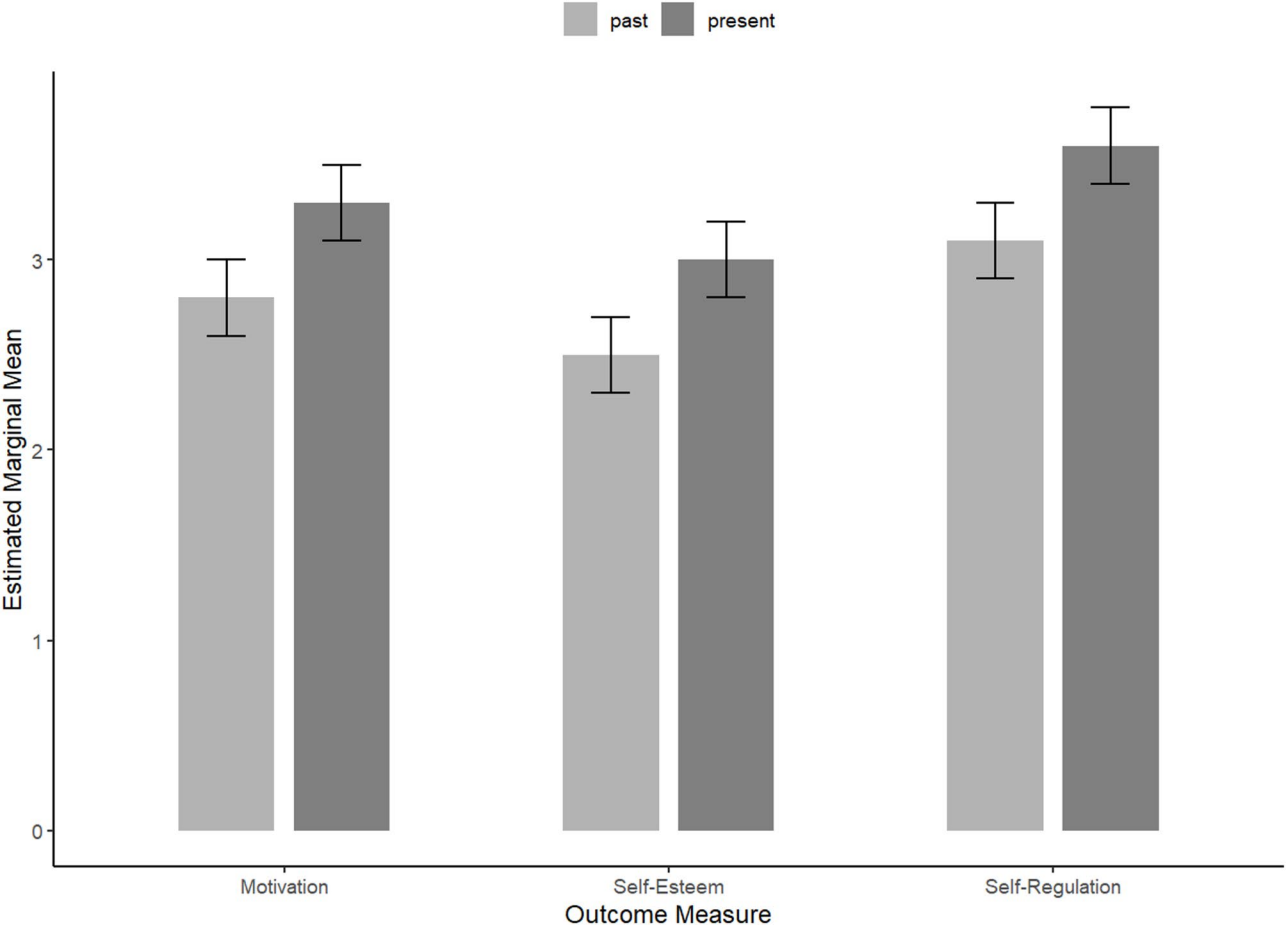
Estimated marginal means of motivation, self-esteem, and self-regulation at past and present time points, controlling for demographic variables. Error bars represent 95% confidence intervals

A linear mixed model was used to examine changes in self-regulation from Past to Present while controlling for gender, age, education, and job status. Results showed a significant effect of time, with participants’ self-regulation scores being higher in the Present condition compared to the Past (*b* = 0.47, SE = 0.07, t(81) = 6.74, *p* <.001), as visualized in Fig. 1. The effect size was large (d = − 0.80), indicating a substantial increase in self-regulation over time (Table 1). None of the demographic variables (gender, age, education, job status) reached statistical significance (*ps* > 0.10), indicating that these factors did not influence the increase in self-regulation over time (Table 1).

**Table 1:** Paired t-test results comparing pre- and post-intervention scores for self-regulation, self-esteem, and motivation

Variable	Group	M	SD	t	df	*p*	95% CI	Cohen's d (Effect Size)	95% CI (d)
Self-Regulation	Past	3.11	0.63	−6.74	81	<.001	[−0.61, −0.33]	−0.80 (Large)	[−1.07, −0.53]
	Present	3.58	0.52						
Self-Esteem	Past	2.47	0.71	−5.80	81	<.001	[−0.55, −0.27]	−0.64 (Medium)	[−0.87, −0.40]
	Present	2.87	0.55						
Motivation	Past	2.67	1.15	−5.87	81	<.001	[−1.08, −0.53]	−0.81 (Large)	[−1.12, −0.50]
	Present	3.48	0.79

**Table 2:** Thematic analysis of intrapersonal system following Internal Cohesion Psychotherapy

Main Theme	Subtheme	Illustrative Quote
Self-Regulation	Cognitive Flexibility	*“Through the process of therapy I went a little deeper*,* I understood that both perceptions and my opinions can change*,* and I can change those thoughts about myself.”* (AS)
	Interpersonal Gains	*“Even in conflicts*,* I now reflect and judge based on what feels healthy. I’ve also become less sensitive because before I was overly sensitive*,* but now I understand and manage it better.”* (EQVS)
Self-Esteem	Empowerment	*“Every time I leave a session and up until the next I am full of positive energy and full of confidence*,* it feels as though the entire world is mine.”* (AH)
	Balanced Self-Perception	*“Now I’ve started to value myself more and see myself in a better light. Not put myself on a pedestal and see something that I am not*,* but I’ve started to see myself in a more down-to-earth manner. Therapy has helped me understand my potential*,* something I haven’t used in the past.”* (AS)
Motivation	Renewed Drive	*“Before therapy*,* I experienced difficult times marked by low motivation. However*,* now therapy transformed my life*,* increasing my desire to engage in new activities and tasks.”* (AGGS)
	Beyond Comfort	*“Psychotherapy has helped me feel more motivated and to make decisions regarding specific situations because before I struggled due to staying in my comfort zone.”* (VSRH)

This table presents the main themes and subthemes identified through the thematic analysis, along with a representative quote for each. Each quote is followed by a participant pseudonym to ensure anonymity.

In addition, the notable improvements in self-regulation are further illustrated though participants’ declarations (Table 2), which manifested in two interrelated subthemes: enhanced cognitive flexibility and interpersonal gains. Initially, participants described becoming more flexible in their thinking, reconsidering rigid beliefs, and adopting new perspectives. For instance, AS reflected on this process, sharing, *“Through therapy*,* I have learned that life is not constantly good*,* you have to face the bad/negative parts and always learn/take away something from those experiences. Many concepts*,* for example*,* I’ve had misconceptions about*,* but through the process of therapy I went a little deeper*,* I understood that both perceptions and my opinions can change*,* and I can change those thoughts about myself.”* Similarly, RH described confronting fears through a shift in perspective, explaining, *“I used to view freedom differently*,* as I was consumed in every aspect and afraid to confront it. Psychotherapy opened my eyes*,* helping me realize that fear doesn’t simply vanish but can be understood*,* accepted*,* and gradually minimized through consistent effort and actions.”* These reflections illustrate how participants developed greater adaptability and resilience by gaining more control over their thinking.

This shift in thinking also had a notable impact on interpersonal functioning, particularly in communication and conflict management. EH explained, *“Surely*,* I can manage different situations better than I used to in the past. At least*,* I’ve managed to solve my own problems. Lately*,* the obstacles I used to have with communication have become easier than they used to be in the past.”* In a similar vein, EQVS described becoming less impulsive and more reflective, stating, *“As a young man*,* I was more impulsive*,* but psychotherapy has helped me give myself time to think*,* which calms me and gives me the courage to face my decisions. While I still dislike rejection*,* I’ve minimized its effect and am learning to manage that feeling. Even in conflicts*,* I now reflect and judge based on what feels healthy. I’ve also become less sensitive because before I was overly sensitive*,* but now I understand and manage it better.”* Together, these narratives demonstrate how improved emotional regulation translates into more adaptive ways of thinking and relating to others.

### ICP post-treatment enhancement of self-esteem

Similar to self-regulation, the linear mixed model revealed a significant improvement in self-esteem from Past to Present, *b* = 0.41, *SE* = 0.07, *t*(81) = 5.80, *p* <.001 (Fig. 1). The effect size was medium (d = − 0.64), indicating a moderate increase in self-esteem over time, as can be seen in Table 1. Controlling for gender, age, education, and job status did not change the size or significance of this effect, and none of the covariates were significant predictors (*ps* > 0.05). Furthermore, these statistical findings are supported by qualitative insights that represent the real-life experiences of changes in self-esteem, manifested in two interrelated subthemes: empowerment and balanced self-perception. The first aspect, empowerment, reflects the increased sense of confidence, agency, and motivation participants experienced following therapy. AH for example, described this transformation sharing, *“Every time I leave a session and up until the next I am full of positive energy and full of confidence*,* it feels as though the entire world is mine.”* This statement illustrates how therapy actively helped clients feel capable and energized, reinforcing their belief in their ability to act and face challenges.

Alongside this growing sense of empowerment, participants also developed a more balanced self-perception, gaining a grounded, realistic, and emotionally aware understanding of themselves. AS highlighted this shift, stating, *“Now I’ve started to value myself more and see myself in a better light. Not put myself on a pedestal and see something that I am not*,* but I’ve started to see myself in a more down-to-earth manner. Therapy has helped me understand my potential*,* something I haven’t used in the past. Surely*,* therapy has helped me get in touch with my emotional side and understand that I have potential/abilities to feel for someone.”* Similarly, AGDK reflected on overcoming a history of low self-esteem, noting, *“Since childhood*,* my self-esteem has been very low because I wasn’t allowed the freedom to express my thoughts*,* leaving me without the confidence to pursue anything. Now*,* however*,* I’ve come to realize that I have value*,* no matter the circumstances.”* These narratives indicate how ICP rebuilds self-esteem by empowering and assisting clients in recognizing their own value.

### Elevation in motivation following ICP treatment

When examining motivation, participants again showed a statistically significant increase in scores at the present point relative to the Past, *b* = 0.80, *SE* = 0.14, *t*(81) = 5.87, *p* <.001 (Fig. 1). Education approached statistical significance (*b* = 0.26, *p* =.064), suggesting a possible trend in which higher education might be associated with higher motivation, though it wasn’t statistically significant. The effect size was large (d = − 0.81), indicating a substantial increase in motivation over time (Table 1). Gender, age, and job status did not have notable effects on motivation (*ps* > 0.20). Moreover, this improvement is reflected in participants’ declarations through two interconnected aspects: renewed drive and moving beyond comfort. They emphasized how therapy reignited their energy to pursue personal and professional goals. For instance, AGGS shared, *“Before therapy*,* I experienced difficult times marked by low motivation. However*,* now therapy transformed my life*,* increasing my desire to engage in new activities and tasks.”* This narrative complements the quantitative findings by underlining how ICP reaffirms the client’s motivation engagement in daily life. At the same time, motivation was also expressed through the courage to step beyond one’s comfort zone. VSRH explained, *“Psychotherapy has helped me feel more motivated and to make decisions regarding specific situations because before I struggled due to staying in my comfort zone.”* Correspondingly, ASVS stated *“I have always wanted to stay in my comfort zone*,* but psychotherapy has helped me become more courageous in expressing my thoughts and encouraged me to change my routine.”* These reflections illustrate how motivation was strengthened not only by renewed energy but also by a greater willingness to embrace change and take proactive steps beyond one’s comfort zone.

## Discussion

This study examined the effectiveness of Internal Cohesion Psychotherapy (ICP) in enhancing the intrapersonal functioning of young clients, specifically their self-regulation, self-esteem, and motivation, using both qualitative and quantitative measures. While previous research highlighted ICP’s role in alleviating anxiety and depression symptoms [34, 38], this study adopts a strengths-based perspective. Results indicate significant improvements across all three dimensions post-treatment, underscoring ICP’s positive impact. This is the first study to integrate both qualitative and quantitative evidence to demonstrate that ICP strengthens self-regulation, enhances motivation, and fosters adaptive self-esteem, providing empirical support for its role in building intrapersonal resilience. These intrapersonal improvements can be explained by some therapeutic techniques used during the ICP session. Several techniques target self-regulation, such as *time awareness journaling*, *emotion diaries*, and *functional scenario exploration*, which help clients monitor their emotions, recognize patterns, and rehearse adaptive responses. For self-esteem, techniques like *honest intra-communication*, *strength-based self-evaluation lists*, and *achievement reflection lists* encourage clients to confront self-critical thoughts while reinforcing positive self-perceptions. Motivation is supported through techniques such as *time-framed visioning*, *the new challenge*, and *purposeful yes or no assessments*, which guide clients in setting realistic goals, making decisions, and sustaining commitment to personal growth. Complementary strategies, including *narrative reconstruction*, *multiple reflections*, and *psychoeducation*, integrate these processes by helping clients reinterpret past experiences and acquire skills for future challenges. Importantly, ICP also incorporates numerous techniques that are not explicitly categorized for any single system or problem; rather, they are applied flexibly depending on the client’s needs, therapeutic goals, and the dynamic interplay between systems. By integrating different techniques within the systems of ICP, therapists enable clients to activate existing strengths and transform maladaptive patterns [35, 39]. Following the rationale of dynamic and bidirectional relations between systems [56], these substantial changes in interpersonal systems have multiple benefits for the client’s life, including their interpersonal and professional relationships.

Previous research has shown that these key psychological factors can be improved by various psychotherapeutic approaches, which is consistent with our findings. For example, through Self-System Therapy, which includes specific strategies and guided psychoeducation that focus on aspects of promotion and prevention, people can improve self-regulation-related challenges [57]. Also, two different systematic reviews found that self-compassion-based therapies, including mindfulness-based interventions [58] and social cognitive interventions [59], significantly improved self-regulation and its components. Additionally, psychotherapeutic approaches for eating disorders were shown to improve self-esteem more so than in control trials [60]. Finally, by emphasizing external elements that are simpler to execute than internal ones, motivational psychotherapy has been demonstrated to be beneficial in increasing clients’ motivation [61]. These approaches that focus on singular dimensions of the intrapersonal system seem to be effective predominantly in that aspect, whereas the ICP utilizes a more integrative approach by aiming to improve multiple dimensions using the client’s strengths and weaknesses in specific dimensions to do so.

Traditional and monotherapeutic approaches tend to value decreasing negative symptoms, increasing the quality of life, and reaching personal goals from the patient’s perspective; they generally overlook the importance of effective coping mechanisms and personal strengths to prevent relapse [62]. In contrast, integrating the principles of Positive Youth Development (PYD) into approaches like Internal Cohesion Psychotherapy (ICP) has been shown to enhance clients’ psychological well-being by strengthening positive dimensions while reducing negative symptoms. This was seen in the qualitative results of this study, where participants reported that their negative mental state, which was caused by low motivation or self-esteem, was significantly improved from ICP sessions and became a positive aspect of functioning. As clients revealed, the enhanced and higher-quality intrapersonal relationship was accompanied by better interpersonal and professional relationships. Following a rational explanation from the client’s perspective, when the clients enhanced their self-regulation, they had a better understanding of others’ needs, higher levels of empathy, and better communication, which led to better social interaction or interpersonal relationships. These findings are supported by other studies, which have shown that increasing self-regulation skills will be associated with increased study time, better academic goals, higher self-discipline and self-motivation, more adaptive stress management, and work organization [63].

In addition, higher motivation and self-esteem were associated with better academic performance, showing the bidirectional relationship and potential pathway for intervention by focusing on positive aspects, rather than just the negative sides of functioning. For example, there are other studies supporting the idea of focusing on positive aspects of functioning, which have shown that more motivation was associated with decreased depression after a year. Specifically, young adults who had positive motivational attitudes were protected against depressive symptoms, and those who were highly motivated were less likely to exhibit signs of feelings of discomfort [64]. Similarly, the significant improvement in self-esteem observed in this study aligns with prior research, such as that from [65], which indicates that having a high sense of self-worth results in being happier, possibly by lessening the negative impacts of stress, even though a causal relationship has not been conclusively shown.

Although results provide strong evidence for the effectiveness of the ICP in reducing depression and anxiety symptoms, some limitations, including sampling, demographic distribution, and the qualitative and cross-sectional nature of the study, must be addressed. Careful consideration must be given to the potential impact of participant self-selection. Individuals who chose to participate in the study may differ systematically from those who did not. For example, they may have been more motivated, more engaged in therapy, or held stronger opinions about their experiences. As a result, this may limit the generalizability of the findings, since the sample may not fully represent the broader population. On a similar note, another aspect of this study that could impact generalizability is the demographic distribution, which had a higher ratio of females to males. This imbalance may have influenced the outcomes, although the existing literature on gender differences in psychotherapy is mixed. Some studies report no substantial differences, while others suggest that considering gender dynamics in therapeutic processes can yield positive results [66, 67]. Given this uncertainty, the predominance of female participants should be acknowledged as a factor that may limit the external validity of the findings. Another key limitation lies in the inherently subjective nature of the qualitative findings, which may introduce interpretive biases. However, this limitation is partially mitigated by the inclusion of quantitative data, which provides more objective evidence of the observed changes. Together, the combination of qualitative and quantitative approaches offers a more comprehensive understanding of the phenomena under study, balancing depth of insight with empirical rigor. The last limitation of this study is the lack of longitudinal data, which would help understand if the effects of psychotherapy are sustainable over time. This limitation could be the focus of future studies exploring ICP or similar interventions. Building on these limitations, future research could explore ICP or similar interventions as well as how PYD approaches affect the mental health of different youth populations. In particular, studies should strive for more gender-balanced samples to clarify whether potential gender dynamics influence the effectiveness of ICP. Randomized controlled trials (RCTs) and comparative studies with other therapeutic approaches can be conducted to further evaluate and enhance the effectiveness of ICP.

## Conclusion

This study provides evidence that Internal Cohesion Psychotherapy (ICP) significantly improves self-regulation, self-esteem, and motivation in youth with mood disorders, aligning with prior research on strengths-based therapeutic approaches. By integrating the principles of Positive Youth Development (PYD), ICP emphasizes the importance of a holistic approach to mental health interventions by focusing on multiple aspects of functions. Participants’ experiences revealed enhanced interpersonal and professional relationships, increased self-worth, and improved coping mechanisms. Future research should examine the sustainability of these outcomes and the broader applicability of ICP in diverse settings and among other diagnoses. These findings reinforce the importance of reframing psychotherapy to include positive development as a primary goal, offering a transformative approach to mental health care.

## Data Availability

The data generated and/or analyzed during this study are available upon request.

## Abbreviations

ACT: Acceptance and commitment therapy
ICP: Internal cohesion psychotherapy
ICQ: Internal cohesion questionnaire
LMM: Linear mixed models
MDD: Major depressive disorder
OCD: Obsessive-compulsive disorder
PYD: Positive youth development
PTSD: Post-traumatic stress disorder
RCTs: Randomized controlled trials
SRQ: Self-regulation questionnaire
SSRQ: Short self-regulation questionnaire
